# Allosteric Inhibition of Factor XIIIa. Non-Saccharide Glycosaminoglycan Mimetics, but Not Glycosaminoglycans, Exhibit Promising Inhibition Profile

**DOI:** 10.1371/journal.pone.0160189

**Published:** 2016-07-28

**Authors:** Rami A. Al-Horani, Rajesh Karuturi, Michael Lee, Daniel K. Afosah, Umesh R. Desai

**Affiliations:** Department of Medicinal Chemistry & Institute for Structural Biology, Drug Discovery and Development, Virginia Commonwealth University, Richmond, Virginia, United States of America; University of Parma, ITALY

## Abstract

Factor XIIIa (FXIIIa) is a transglutaminase that catalyzes the last step in the coagulation process. Orthostery is the only approach that has been exploited to design FXIIIa inhibitors. Yet, allosteric inhibition of FXIIIa is a paradigm that may offer a key advantage of controlled inhibition over orthosteric inhibition. Such an approach is likely to lead to novel FXIIIa inhibitors that do not carry bleeding risks. We reasoned that targeting a collection of basic amino acid residues distant from FXIIIa’s active site by using sulfated glycosaminoglycans (GAGs) or non-saccharide GAG mimetics (NSGMs) would lead to the discovery of the first allosteric FXIIIa inhibitors. We tested a library of 22 variably sulfated GAGs and NSGMs against human FXIIIa to discover promising hits. Interestingly, although some GAGs bound to FXIIIa better than NSGMs, no GAG displayed any inhibition. An undecasulfated quercetin analog was found to inhibit FXIIIa with reasonable potency (efficacy of 98%). Michaelis-Menten kinetic studies revealed an allosteric mechanism of inhibition. Fluorescence studies confirmed close correspondence between binding affinity and inhibition potency, as expected for an allosteric process. The inhibitor was reversible and at least 9-fold- and 26-fold selective over two GAG-binding proteins factor Xa (efficacy of 71%) and thrombin, respectively, and at least 27-fold selective over a cysteine protease papain. The inhibitor also inhibited the FXIIIa-mediated polymerization of fibrin *in vitro*. Overall, our work presents the proof-of-principle that FXIIIa can be allosterically modulated by sulfated non-saccharide agents much smaller than GAGs, which should enable the design of selective and safe anticoagulants.

## Introduction

Thrombotic disorders, such as venous thromboembolism (VTE), stroke, myocardial infraction and other indications, constitute a major health burden for most countries. Even with the current repertoire of antithrombotics used in the clinic, a large number of patients develop recurrent VTE and/or suffer from major long-term complications [1–3]. A growing body of literature suggests that VTE and cancer are strongly linked. Patients with cancer have at least 4-fold increased risk for VTE and patients with VTE are at higher risk for developing cancer [4]. A range of anticoagulants is used in the clinic today to treat and prevent episodes of thrombosis including the antithrombin-based agents (heparins), vitamin K-based agents (coumarins), and the newer direct thrombin or factor Xa inhibitors [5]. Despite their success, each agent carries a number of drawbacks, of which a common risk is internal bleeding, which can be life-threatening [6]. Thus, approaches that seek completely different routes of engineering anticoagulant activity, with a promise of reducing bleeding complications, are important to develop.

Among these newer approaches is the concept of targeting factor XIIIa (FXIIIa), which catalyzes the final step in the coagulation process by cross-linking the α- and γ-chains of fibrin. FXIIIa is the only enzyme of the coagulation system that is not a serine protease. It is a thiol-based transglutaminase that conjugates two substrates in a process that involves formation of a thio-ester-based acyl enzyme intermediate [7,8]. This intermediate, instead of being acted upon by a molecule of water to afford a cleaved product as for serine proteases, is acted upon by a nearby amine (Lys residue) of the second substrate to give the coupled product. Absence of this lysine can induce hydrolysis of the thio-ester intermediate resulting in an incomplete conjugation step [7,8].

FXIIIa can also cross-link α_2_-plasmin inhibitor to fibrin, which helps protect the newly formed clot from plasmin-mediated fibrinolysis [9]. This contributes to the clot’s higher biochemical and biophysical stability. In addition, the size of thrombus is also dependent on FXIIIa action [10, 11]. In fact, a model of ligation-mediated thrombosis of the mouse inferior vena cava showed that FXIII-deficiency resulted in a significant reduction (~50%) in thrombus weight arising from a reduced RBC content [10, 11]. This implies that chemical inhibition of FXIIIa may lead to weaker and smaller thrombus formation, which may be more susceptible to fibrinolysis. In fact, tridegin, a peptide from leech *Haementeria ghilianii* that inhibits FXIIIa, does reduce clot strength and stability [12–16]. This, when coupled with the observation that heterologous FXIII gene knockout in the mouse is not associated with signs of excessive bleeding [10,17,18], suggests that the transglutaminase FXIIIa may serve as a promising therapeutic target to prevent and/or treat VTE and other thrombotic disorders.

Despite the apparent advantages, very few FXIIIa inhibitors have been reported in literature. Tridegin is the most studied inhibitor [12–16]. It is a 66-mer polypeptide that will be challenging to transform into a small molecule scaffold. Small molecule inhibitors of FXIIIa reported to date include active site-directed irreversible agents [19], imidazolium salts, [20] thiadiazoles [21] and cyclopropenoids [22]. These, and other miscellaneous agents [19], were developed as early leads and/or probes of FXIIIa mechanism, and appear to have not been followed up with advanced studies.

*A priori*, the double-displacement mechanism offers more opportunities of interfering with the catalytic cycle because two substrates have to be engaged in succession [23]. Such fine regulation is likely to be highly susceptible to minor changes in conformation of the active site. We thus hypothesized that allosteric modulation of FXIIIa’s active site should be achievable. In principle, allosteric inhibition can offer two major advantages including 1) higher target specificity and 2) better regulation of activity in comparison to orthosteric inhibitors [24–30]. Of these, the latter point is especially important for FXIIIa because complete elimination of this enzyme has been known to result in bleeding diathesis [31–33], whereas partial reduction in its activity results in only minor adverse effects [10, 17, 18]. As demonstrated for thrombin earlier [34–38], allostery is the only process for engineering partial inhibition. Therefore, discovering allosteric inhibitors of FXIIIa is likely to open up a major route to novel molecules that could eventually afford safer anticoagulants.

**Fig 1:**
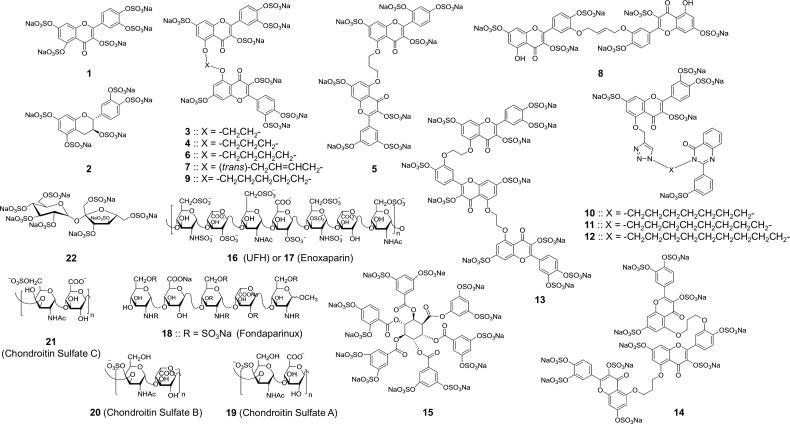
The library of sulfated GAGs and NSGMs. Molecules in this library possessed structural diversity at multiple levels, e.g., different core scaffolds, number of sulfate groups, and position of sulfate groups. NSGMs included five distinct chemical classes of pentasulfated monomeric flavonoid scaffolds (**1**–**2**), hexa- and octa- sulfated dimeric flavones (**3**–**9**), pentasulfated dimeric flavone-quinazolinones (**10**–**12**), undecasulfated trimeric flavones (**13** and **14**), and dodecasulfated hexabenzoyl inositol derivative (**15**). GAGs comprised of unfractionated heparin (UFH, **16**), enoxaparin (**17**), and chondroitin sulfate A-C (**19**–**21**) in addition to the octasulfated pentasaccharide fondaparinux (**18**) and the octasulfated disaccharide sucrose octasulfate (**22**).

With this objective, we tested a library of 22 variably sulfated glycosaminoglycans (GAGs) and their nonsaccharide mimetics against human FXIIIa (Fig 1). An undecasulfated quercetin trimer **13** was identified as a promising inhibitor that displays allosteric inhibition mechanism. The agent inhibited the FXIIIa-mediated crosslinking of fibrin suggesting its potential as a promising lead. Overall, this work presents the first mechanistic evidence that FXIIIa can be allosterically modulated by appropriate small molecules that may display effective and safe anticoagulation properties.

## Results and Discussion

### Rationale for Screening Sulfated Molecules against Human FXIIIa

**Fig 2:**
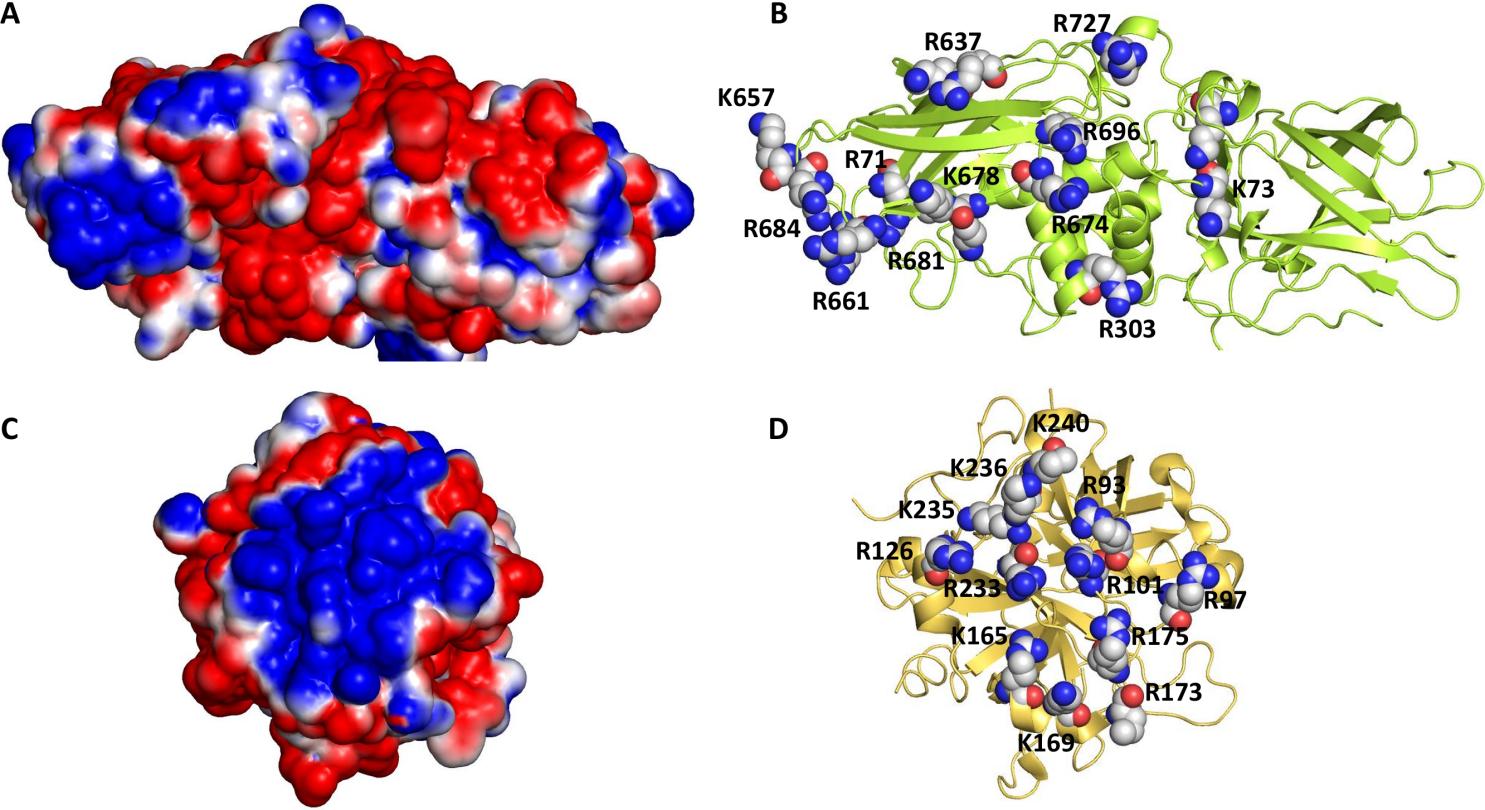
The putative anion-binding allosteric site of human FXIIIa. (A) The electrostatic potential of the surface exposed anion-binding site of FXIII (PDB ID: 1GGU). (B) The basic residues in the site are shown as spheres. The residues matching the heparin-binding site of transglutaminase are K61, K73, R303, and K678. (C) The electrostatic potential of human thrombin is shown (PDB ID: 1XMN). (D) The basic residues of thrombin’s exosite 2 are shown in spheres. Positive and negative potentials are colored in blue and red, respectively.

Plasma FXIII is a heterotetramer of two A and two B subunits (FXIIIA_2_B_2_). The A subunit (FXIII-A) contains the catalytic domain and the B subunit (FXIII-B) serves as a carrier and regulatory protein. FXIIIA_2_B_2_ is activated by thrombin through cleavage of Arg37–Gly38 bond of A subunits, which is followed by dissociation of the B subunits in the presence of Ca^2+^. The resulting FXIIIa has a reactive Cys314 residue in the active site, which cross-links Lys and Gln residues of fibrin α- and γ- chains leading to a three-dimensional, insoluble fibrin network [9, 39]. The A subunit of FXIIIa has a relatively high number of Lys and Arg residues that are clustered together on the surface potentially forming an anion-binding exosite (Fig 2A and 2B), which is located ~16–24 Å from the enzyme active site. Although this exosite bears no homology with thrombin’s exosite 2, which interacts with heparin (Fig 2C and 2D) [40], it may potentially serve as an allosteric site to which the sulfated GAGs and/or non-saccharide mimetics of GAGs may bind and inhibit FXIIIa. Interestingly, a site on a homologous enzyme, transglutaminase 2, was recently identified as the heparin binding site and this site appears to be important for enzyme function [41,42]. A characteristic feature of these sites is that they contain multiple Arg/Lys residues, which most probably interact with multiple sulfate groups on heparin and related GAGs. Yet, these Arg/Lys residues are distinct in terms of the location and orientation. Further, these sites differ in terms of their hydrophobic sub-sites [41–43], which may serve as recognition elements for sulfated nonsaccharide GAG mimetics (NSGMs), as demonstrated earlier [37, 38, 44–46]. This led to our hypothesis that one of the GAGs and/or NSGMs, which electrostatically and/or hydrophobically complements the anion-binding site, may transmit binding energy to the active site and function as selective allosteric inhibitor(s) of FXIIIa.

To test this hypothesis, we studied a focused library of 22 variably sulfated GAGs and sulfated NSGMs (Fig 1). NSGMs included five distinct chemical classes of pentasulfated monomeric flavonoids (**1** and **2**), hexa- and octa-sulfated dimeric flavones (**3**–**9**), pentasulfated dimeric flavone-quinazolinones (**10**–**12**), undecasulfated trimeric flavones (**13** and **14**), and dodecasulfated hexabenzoyl inositol derivative (**15**). The group of GAGs included natural sequences of unfractionated heparin (UFH, **16**), enoxaparin (**17**), and chondroitin sulfate A-C (**19** and **21**), as well as synthetic molecules such as the octasulfated pentasaccharide fondaparinux (**18**) and the disaccharide sucrose octasulfate (**22**). Thus, the focused library afforded excellent diversity of core scaffolds, orientation of sulfate groups and sulfation level so as to ensure higher probability of discovering allosteric inhibitors.

### Inhibition of Human FXIIIa by the Library of Sulfated Molecules

The library of sulfated molecules was screened for inhibition of human FXIIIa using a modified bisubstrate, fluorescence-based transglutamination assay, as described earlier [47]. Dansylcadaverine and *N*,*N*-dimethyl-casein were used as two substrates, which upon FXIIIa-dependence conjugation show a marked increase in fluorescence at 550 nm (λ_EX_ = 360 nm). Initial screening was performed at 200 μM NSGM concentration (**1**–**15**) or 50–1110 μM GAG concentration (**16–22**) (Table 1). At these levels, none of GAGs inhibited FXIIIa by more than 5%. Sucrose octasulfate (**22**) inhibited FXIIIa by about 17 ± 2%. Interestingly, NSGMs displayed varying inhibition potential. Pentasulfated **1** and **2**, a flavone and flavanol monomers, respectively, inhibited FXIIIa by ~30%, whereas polysulfated, homo or heterodimeric flavonoids **3**–**12** exhibited inhibition levels of 53 to 89%. Of these, the octasulfated homodimeric flavone **6** was the most efficacious (89% inhibition). Trimeric scaffolds **13** and **14** inhibited FXIIIa by 94% and 29%, respectively. Interestingly, the dodecasulfated hexabenzoyl inositol derivative **15** inhibited FXIIIa only 55% at 200 μM concentration. To identify promising NSGMs from these, screening was performed at a lower concentration (20 μM). The results showed **3**, **6**, **7** and **10** induced 27 to 40% inhibition, whereas **13** inhibited FXIIIa by 55% (Table 1). Thus, the molecule that exhibited the most potent inhibition at 20 μM was the flavone trimer **13**.

**Table 1:** Inhibition Percent of Human FXIIIa by Sulfated Small Molecules.^a^

Molecule	No. of sulfates	FXIIIa inhibition at 200 μM (%)	FXIIIa inhibition at 20 μM (%)
1	5	31 ± 4^b^	ND^c^
2	5	32 ± 6	ND
3	8	65 ± 3	26 ± 2
4	8	67 ± 5	ND
5	8	76 ± 4	ND
6	8	89 ± 5	40 ± 3
7	8	58 ± 4	31 ± 3
8	6	84 ± 5	ND
9	8	53 ± 5	ND
10	5	88 ± 7	27 ± 1
11	5	81 ± 7	ND
12	5	80 ± 6	ND
13	11	94 ± 4	55 ± 2
14	11	29 ± 6	ND
15	12	55 ± 7	ND
16	Variable	~2 (at 250 μM)	ND
17	Variable	~3 (at 1110 μM)	ND
18	8	~0 (at 360 μM)	ND
19	Variable	~1 (at 50 μM)	ND
20	Variable	~0 (at 50 μM)	ND
21	Variable	~0 (at 50 μM)	ND
22	8	17 ± 2	ND

^a^ The percentages of inhibition of human FXIIIa by different sulfated molecules were monitored by a transglutamination assay using a bisubstrate-based fluorescence spectroscopy (λ_Ex_ = 360 nm and λ_Em_ = 550 nm) in 50 mM TrisHCl (pH 8.0) containing 10 mM CaCl_2_ and 100 mM NaCl at 37°C. See Materials and Methods for details.

^b^ Errors represent ± 1 S.E.

^c^ Not determined.

**Fig 3:**
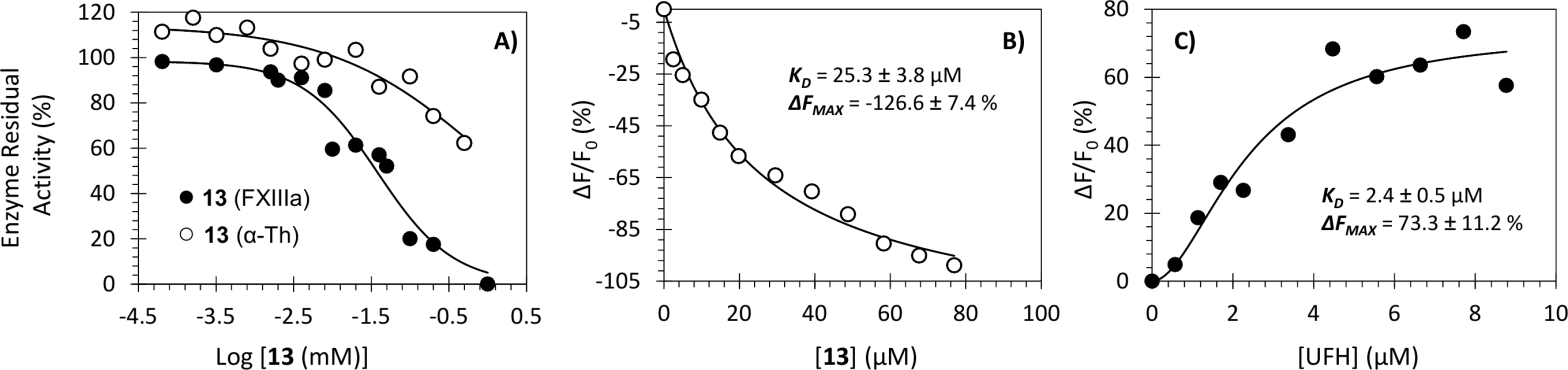
Interaction of human FXIIIa and α-thrombin (α-Th) with NSGM 13 and UFH. (A) The inhibition of FXIIIa (●) and α-Th (○) by NSGM **13** was measured spectrofluorometrically through a bisubstrate, fluorescence-based transglutamination assay (FXIIIa) or chromogenic substrate assay (α-Th) at pH 7.4/8.0 and 37°C. Solid lines represent sigmoidal fits to the data to obtain *IC*_*50*_, *HS*, *Y*_*M*_, *and Y*_*O*_ using Eq 1. (B) Spectrofluorometric measurement of the affinity of human FXIIIa for inhibitor **13** at pH 8.0 and 37°C using the intrinsic tryptophan fluorescence (λ_EM_ = 348 nm, λ_EX_ = 280 nm). Solid lines represent nonlinear regressional fits using quadratic Eq 2. (C) Spectrofluorimetric measurement of the affinity of human FXIIIa for UFH at pH 8.0 and 37°C using the intrinsic tryptophan fluorescence (λ_EM_ = 348 nm, λ_EX_ = 280 nm). Solid lines represent nonlinear regressional fits using the standard Hill Eq 3. See details in Materials and Methods.

To measure the potency and efficacy of inhibitor **13**, the dose-dependence of FXIIIa inhibition was evaluated using the logistic Eq 1 (see Table 2). The potency of inhibition refers to the *IC*_*50*_ (x-axis), whereas the efficacy refers to the net change in residual FXIIIa activity (*ΔY*) (y-axis). Representative inhibition profiles are shown in Fig 3A. Molecule **13** inhibited FXIIIa with an *IC*_*50*_ of 36.2 μM and efficacy of 98%. These inhibition parameters were independent of enzyme concentration (see S1 Table). The structurally related trimer **14** inhibited FXIIIa with a much weaker potency (118.0 μM) and an almost equivalent efficacy (93%). Iodoacetamide, a nonselective inhibitor of thiol-containing enzymes, was used as a positive control. It inhibited human FXIIIa with an *IC*_*50*_ of 2.9 μM (efficacy = ~100%, Table 2).

**Table 2:** Inhibition Profiles of Human Factor XIIIa (FXIIIa), Human α-Thrombin (α-Th), Human Factor Xa (FXa), and Papain by Iodoacetamide (IAA) and the NSGMs 13 and 14.^a^

Inhibitor	IC_50_ (μM)	HS	ΔY (%)
IAA (FXIIIa)	2.9 ± 0.4^b^	0.8 ± 0.2	105 ± 10
13 (FXIIIa)	36.2 ± 4.5	0.9 ± 0.2	98 ± 11
14 (FXIIIa)	118.0 ± 49.9	1.1 ± 0.5	93 ± 30
13 (α-Th)	>930	ND^c^	ND
13 (FXa)	313.5 ± 119.6	0.9 ± 0.4	71 ± 22
13 (Papain)	>1000	ND	ND

^a^ The *IC*_*50*_, *HS*, and *∆Y* values were obtained following non-linear regression analysis of direct inhibition of FXIIIa, α-Th, FXa, or papain in appropriate TrisHCl buffers of pH 7.4–8.0 at 37°C containing appropriate concentrations of NaCl and CaCl_2_. See Materials and Methods for details.

^b^ Errors represent ± 1 S.E.

^c^ Not determined.

We also evaluated NSGM **13** against guinea pig transglutaminase (gTG), a very closely related enzyme. NSGM **13** inhibited gTG in a comparable manner with an *IC*_*50*_ of 23.5 μM and an efficacy of 87% (Table 2). Although gTG is not relevant for application with regard to humans, it would be important to engineer an analog of **13** that displays higher selectivity against human transglutaminases.

### Structure-Activity Relationship of Human FXIIIa Inhibition

To develop a better understanding for structural elements required for FXIIIa inhibition by these inhibitors, we closely re-examined their structures and their corresponding inhibition profiles. The monomeric flavonoids were the weakest inhibitors among all NSGMs irrespective of the central moiety being either unsaturated (**1**) or saturated (**2**). Coupling of two flavone moieties using alkylene linkers to form dimeric scaffolds generally improved the inhibition potential by at least 2-fold. Within this category, increasing the linker length from 2-atom (**3**) to 4-atom (**6**) enhanced the inhibition efficacy from 65% to 89% (at 200 μM) and from 26% to 40% (at 20 μM). Yet, a trans double bond (**7**) or a longer linker (5-atom, **9**) was detrimental. This suggests that the 4-atom linker appears to be an optimal length for the 5–5-linked dimeric NSGMs. Interestingly, shifting the sulfate group from position-3ˋ (dimer **7**) to position-2ˋ (dimer **5**) increased the inhibition efficacy by ~10%. Moreover, the position of linker chosen for dimerization also appears to be important. Flavone dimer **7** (5─5 coupled) displayed lower efficacy than dimer **8** (3ˋ─3ˋ coupled) despite the fact that **7** is an octasulfated NSGM, whereas **8** is a hexasulfated agent.

To enhance the possibility of more promising inhibitors, we coupled the flavone moiety with the quinazolinone moiety, which resulted in three NSGMs **10**–**12** having different linker lengths. Although the three molecules were only pentasulfated, they exhibited ≥80% FXIIIa inhibition at 200 μM concentration. NSGM **10** was the most potent; however, it failed to induce more than 27% inhibition at 20 μM concentration. It is important to mention here that several disulfated homodimeric quinazolinone–based NSGMs (structures are not shown) were also screened against FXIIIa without much success. This implied that the flavone moiety was the favored scaffold for FXIIIa inhibition. This led to the study of flavone homotrimer **13**, which is the best inhibitor identified in this study. Even here, the linker length (propylenic *versus* ethylenic) was found to be crucial. Homotrimer **14** displayed reduced inhibition potency in comparison to homotrimer **13**.

Previous studies have shown that polyanion-binding proteins do not necessarily rely on high sulfation level of their ligands but also on the 3-dimentional orientation of the sulfate groups. For example, factor XIa was found to preferentially recognize globular NSGMs [45], while antithrombin interacted better with linear sulfated molecules [46]. To test the impact of sulfate group orientation on FXIIIa inhibition, we studied NSGM **15**. This molecule contains similar number of sulfate groups as compared to **13** but has a more globular shape. Inhibitor **15** was about 2-fold less active than the trimer **13** at 200 μM concentration.

Finally, the backbone of FXIIIa inhibitors appears to be very critical. Whereas most NSGMs exhibit some inhibition potential, the GAGs (**16**–**22**), which are perhaps more highly sulfated, display very poor inhibition characteristics (concentrations <250 μM). UFH **16** has no direct FXIIIa inhibition property despite the fact that it was found to bind to FXIIIa fairly well (*K*_*D*_ of 2.4 μM, Fig 3C). Inhibitor **13** was found to bind to FXIIIa with a *K*_*D*_ value of ~25 μM (Fig 3B). Overall, these results highlight the significance of the aromatic scaffold of NSGMs for inhibiting human FXIIIa.

### Mechanism of FXIIIa Inhibition by NSGM 13

**Fig 4:**
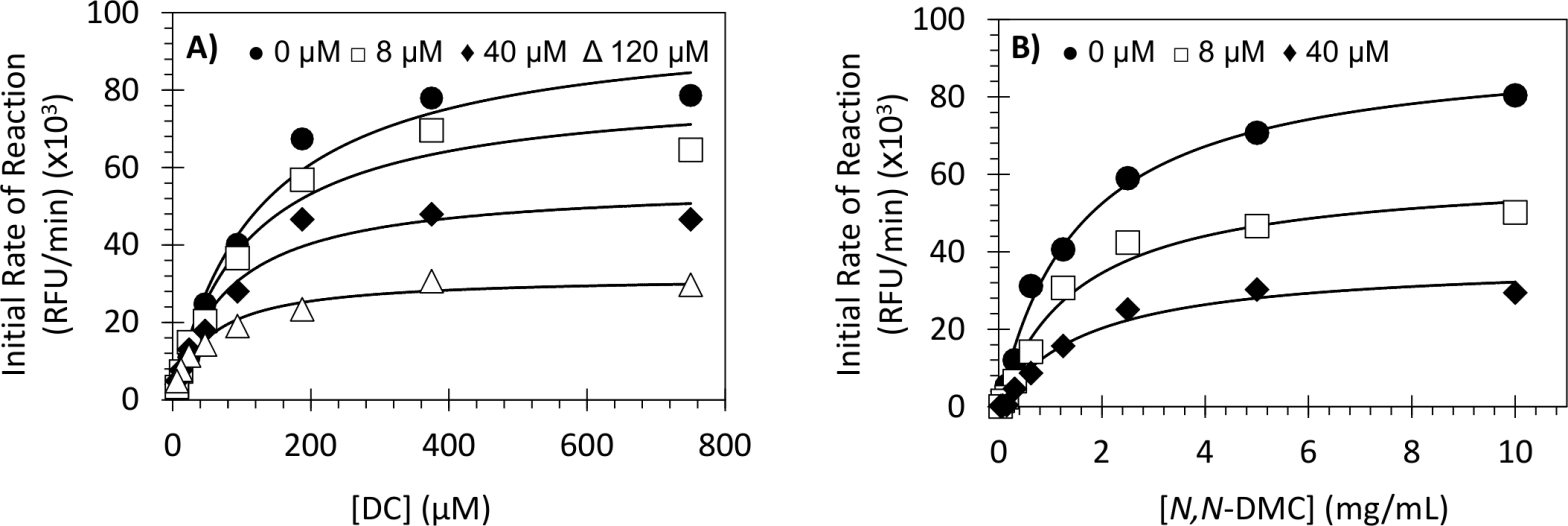
Michaelis−Menten kinetics of dansylcadaverine and *N*,*N*-dimethylcasein conjugation by human FXIIIa in the presence of NSGM 13. The initial rate of conjugation at (A) various dansylcadaverine concentrations (0–750 μM) and fixed dimethylcasein concentration (5 mg/mL) or (B) various dimethylcasein concentrations (0–5 mg/mL) and fixed dansylcadaverine concentration (250 μM) was measured spectrofluorometrically in pH 8.0 buffer at 37°C. Solid lines represent nonlinear regressional fits to the data by the standard Michaelis− Menten Eq 4 to yield *K*_*M*_ and *V*_*MAX*_. See details in Materials and Methods.

To understand the basis for **13**’s FXIIIa inhibitory potential, the kinetics of dansylcadaverine conjugation with dimethylcasein by FXIIIa was studied with and without the inhibitor using the bisubstrate fluorescence assay [47]. At a fixed concentration of dimethylcasein (5 mg/mL), the initial rate of conjugation increased in a hyperbolic manner with increasing concentration of dansylcadaverine (Fig 4A) in the presence as well as absence of inhibitor **13**. Analysis using the Michaelis equation led to *K*_*M*_ values for dansylcadaverine of 123.2 μM and 27.3 μM in the absence and presence of **13**, respectively (Table 3). This represents an increase of ~4.5-fold in dansylcadaverine binding affinity in the presence of **13** at 120 μM. Likewise, the *V*_*MAX*_ also decreased ~3-fold in the presence of the inhibitor.

**Table 3:** Conjugation of Dansylcadaverine (DC) and *N*,*N*-Dimethylcasein (*N*,*N*-DMC) by Human FXIIIa in the Presence of NSGM 13.^a^

[13] (μM)	[N,N-DMC] (mg/mL)	[DC] (μM)	*K*_*M*_	*V*_*MAX*_ (RFU/min) (x10^3^)	*kcat* (1/s) (x10^3^)	*kcat/K*_*M*_ (1/M s) (x10^7^)
0	5	0–750	123.2 ± 25.6^b^ μM	98.4 ± 7.0	54.7 ± 3.8	44.4 ± 9.7
8	5	0–750	105.0 ± 23.2 μM	81.1 ± 5.9	45.1 ±3.3	43.0 ± 10.0
40	5	0–750	77.5 ± 18.6 μM	55.9 ± 4.1	31.1 ± 2.3	40.2 ± 10.1
120	5	0–750	27.3 ± 6.9 μM	32.2 ± 2.0	17.7 ± 0.9	33.4 ± 6.2
0	0–5	250	1.6 ± 0.2 mg/mL	93.5 ± 3.8	51.9 ± 2.1	71.1 ± 9.3
8	0–5	250	1.5 ± 0.3 mg/mL	61.0 ± 4.5	33.9 ± 2.5	49.9 ± 10.9
40	0–5	250	1.7 ± 0.4 mg/mL	37.7 ± 3.1	20.9 ± 1.7	27.1 ± 6.7

^a^ *K*_*M*_ and *V*_*MAX*_ values of the two substrates (N,N-DMC: N,N-Dimethylcasein; DC: Dansylcadaverine) conjugation by human FXIIIa (0.03 μM) were measured using fluorescence spectroscopy (λ_Ex_ = 360 nm and λ_Em_ = 550 nm) in 50 mM TrisHCl (pH 8.0) containing 10 mM CaCl_2_ and 100 mM NaCl at 37°C. RFU indicates relative fluorescence units. Average MWt of casein used is 22 kD.

^b^ Error represents ±1 SE.

To derive kinetic constants for dimethylcasein, we used a fixed, high concentration of dansylcadaverine (250 μM) and studied the initial rate of conjugation, as described above. As expected, a hyperbolic profile was observed with increasing concentration of dimethylcasein (0–5 mg/mL) (Fig 4B) from which the *K*_*M*_ and *V*_*MAX*_ were calculated. The *K*_*M*_ for dimethylcasein in the absence of **13** was measured to be 1.6 mg/mL, which essentially remained invariant in the presence of the inhibitor at 40 μM (Table 3). In contrast, *V*_*MAX*_ decreased ~2.5-fold in the presence of **13** at 40 μM.

It is important to note that FXIIIa-mediated reaction is a bisubstrate conjugation reaction and its interaction with **13** is sensed differently by the two substrates. Whereas the K_M_ of dansylcadaverine decreased upon **13** binding, it did not change for *N*,*N*-dimethylcasein. Yet, for both substrates, there was a decrease in the V_MAX_ with the inhibitor concentration. This alludes to an allosteric process. In these experiments inhibitor **13** was used at varying levels, of which the highest concentration ensured that FXIIIa was essentially fully saturated and inhibited. Thus overall, these results indicate that **13** acts as an uncompetitive inhibitor with respect to the smaller substrate, i.e. dansylcadaverine, and as a noncompetitive inhibitor with respect to the larger substrate, i.e. dimethylcasein.

While for uncompetitive inhibition, the inhibitor binds to FXIIIa-substrate complex only, an ideal noncompetitive inhibition requires equal preference of the inhibitor for both enzyme and enzyme–substrate complex. Because dansylcadaverine is the first substrate to engage FXIIIa, the uncompetitive mechanism suggests simultaneous engagement of both the substrate and the inhibitor by the enzyme. The reduction in rate of this reaction is likely to be conformational change in the active site, which most probably carries forward to the subsequent step without much change in the affinity of dimethylcasein resulting in a non-competitive kinetics. Although plausible, this explanation should be considered hypothetical and detailed kinetic evaluation would be necessary to elucidate the parameters of this ping-pong process. Yet, both mechanisms have to arise from allosteric binding of inhibitor **13** to FXIIIa, which induces dysfunction in its catalytic apparatus. Thus, NSGM **13** is the first allosteric modulator of FXIIIa and likely to open up a major route in the design of FXIIIa inhibitors to treat associated pathologies.

### Selectivity of Inhibition by 13

**Fig 5:**
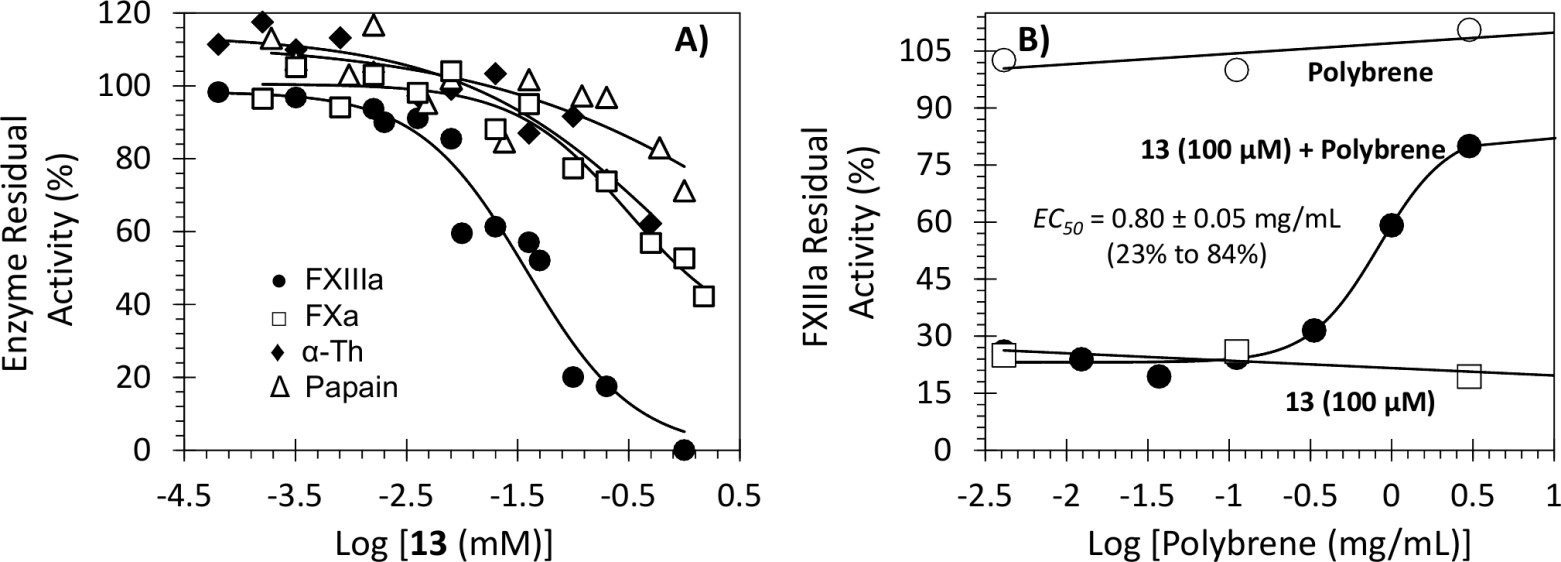
Relative inhibition potency and reversibility of NSGM 13 toward human FXIIIa and related enzymes. (A) Residual activity (%) of four enzymes in the presence of inhibitor **13**. The inhibition of FXIIIa (●), factor Xa (□), thrombin (♦), and papain (Δ) by inhibitor **13** was studied by the corresponding assays at pH 7.4 and 37 °C as described in the Materials and Methods. Solid lines represent the sigmoidal dose−response fits (Eq 1) to the data to obtain the values of *IC*_*50*_, *ΔY*, and HS. (B) Reversibility assay was performed at inhibitor **13** concentration of 100 μM (●) by adding increasing concentration of polybrene (0–3 mg/mL). Shown also the residual FXIIIa activity in the presence of only polybrene (○) or only inhibitor **13** (100 μM) (□). Solid lines represent the sigmoidal fits to the data to obtain *IC*_*50*_ *(or EC*_*50*_*)*, *HS*, *Y*_*M*_, and *Y*_*O*_ using Eqs 1 or 5, as described in the Materials and Methods.

One of the reasons behind targeting an allosteric site on human FXIIIa was to achieve selectivity of inhibition over closely related enzymes. To demonstrate this feature, hydrolysis of appropriate small tripeptidic chromogenic substrates of thrombin and factor Xa, two GAG-binding proteins that play major roles in coagulation, was measured at pH 7.4 and 37°C (Fig 5A). The *IC*_*50*_ values of inhibitor **13** against thrombin and factor Xa were >930 μM and 313.5 μM, respectively (Table 2) suggesting a selectivity index of at least 26-fold and 9-fold, respectively. Additionally, inhibition of factor Xa by **13** displayed an efficacy of 71% as opposed to nearly 100% displayed against FXIIIa. This is important as it further alludes to the allosteric nature of NSGMs [48]. This also suggests that inhibitor **13** could be possibly modified so as to engineer enhanced selectivity and partial inhibition (<100%) [48]. Finally, we studied **13** inhibition of papain, a related cysteine protease. NSGM **13** demonstrated a selectivity index of at least 27-fold over papain (Table 2). Thus overall, the allosteric inhibitor **13** displays a good selectivity.

### Reversibility of Inhibition by 13

Because NSGMs were developed as alternatives to GAGs [49], we hypothesized that inhibition of FXIIIa by NSGM **13** is most probably driven by electrostatic interactions between sulfate groups and their counterparts Arg and Lys on FXIIIa. Therefore, we evaluated polybrene as a rapid antidote of the binding of NSGM **13** to FXIIIa. Polybrene is a quaternary amine-based cationic polymer, which is traditionally used to probe electrostatic interactions of GAGs with their binding proteins [50]. Human FXIIIa was first treated with a high concentration of inhibitor **13** (100 μM) and the recovery of FXIIIa activity by polybrene studied spectro-fluorometrically, as described above at pH 8.0 and 37°C (Fig 5B). The effective concentration of polybrene to restore up to 85% of the original enzyme activity (*EC*_*50*_) were calculated and found to be 0.80 ± 0.05 mg/mL. It is important to emphasize here that polybrene did not affect FXIIIa activity as measured by the bisubstrate fluorescence assay. Thus, this class of allosteric inhibitors, in particular NSGM **13**, appear to interact with FXIIIa through ionic interactions and its inhibition is rapidly reversed by polybrene.

### Effect of Inhibitor 13 on FXIIIa-Mediated Fibrin Polymerization

**Fig 6:**
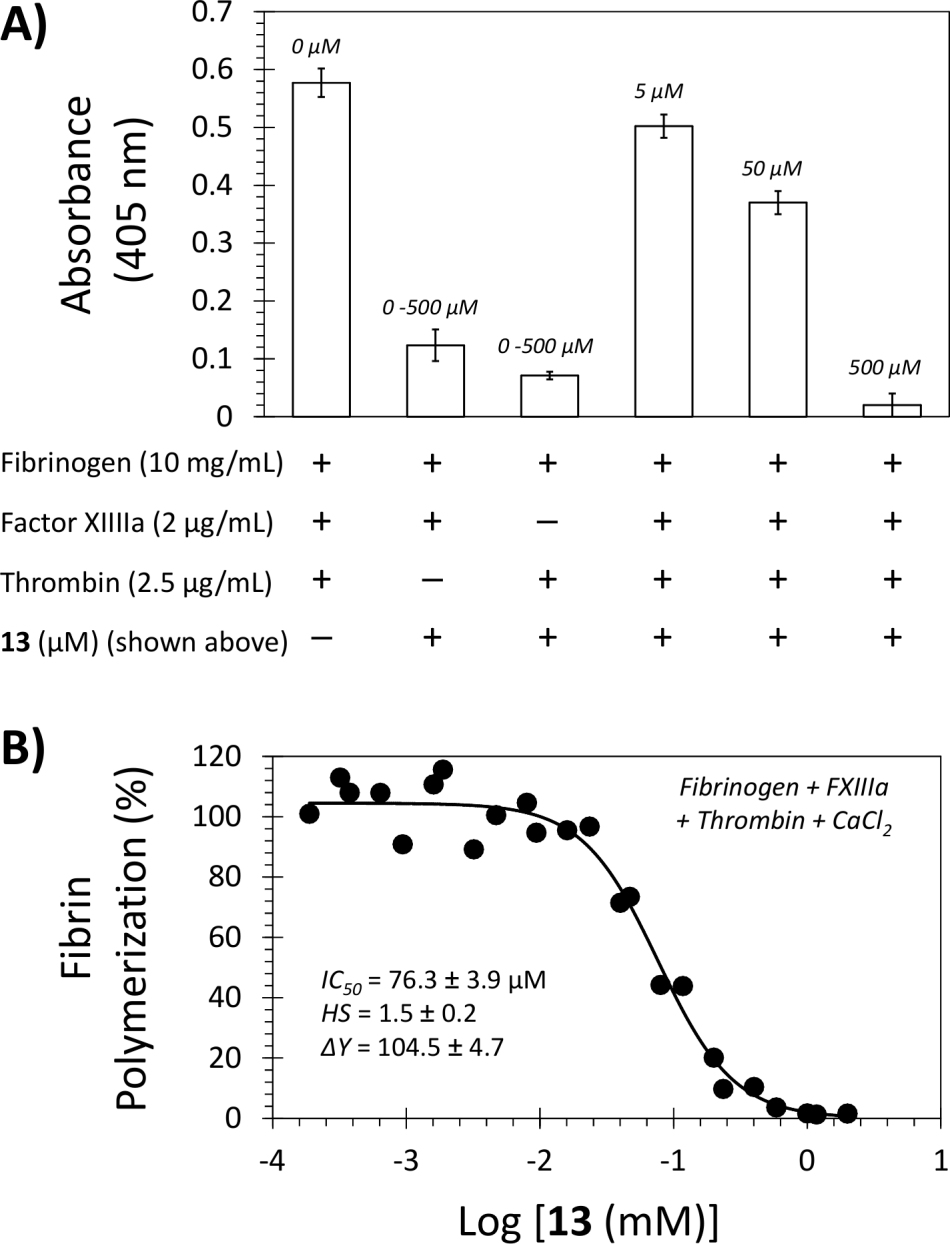
Effect of NSGM 13 on FXIIIa-mediated fibrin crosslinking. (A) The formation of crosslinked fibrin upon addition of aqueous solution of thrombin (2.5 μg/mL) to aqueous solution of fibrinogen (10 mg/mL) and human FXIIIa (2.0 μg/mL) was measured spectrophotometrically at 405 nm and 25°C. The reaction was carried out in 50 mM TrisHCl buffer of pH 7.4 containing 10 mM CaCl_2_ and in the presence or absence of thrombin, FXIIIa, or NSGM **13** (5, 50, and 500 μM). (B) Relative fibrin crosslinking (%) of a solution containing fibrinogen (10 mg/mL), FXIIIa (2.0 μg/mL), thrombin (2.5 μg/mL), and 10 mM CaCl_2_ in the presence of various concentrations of NSGM **13** was determined by measuring the absorbance of each well at 405 nm, pH 7.4, and 25°C. Solid lines represent sigmoidal fits to the data to obtain *IC*_*50*_, *HS*, *Y*_*M*_, and *Y*_*O*_ using Eq 1. See details in Materials and Methods.

To assess the impact of inhibitor **13** on the primary physiologic function of FXIIIa, we used a modified assay in which we monitored cross-linked fibrin formation following activation of fibrinogen by thrombin in the presence of human FXIIIa. The reaction was carried out in 50 mM TrisHCl buffer of pH 7.4 containing 10 mM CaCl_2_. The formation of the crosslinked fibrin was monitored spectrophotometrically at 405 nm and 25 °C. As depicted in Fig 6A, in the presence of 5, 50, and 500 μM **13** fibrin formation decreased dose-dependently to about 87 ± 5%, 64 ± 4%, and 3.5 ± 3.5%, respectively. This suggests that NSGM **13** inhibits fibrin polymerization *in vitro*. Likewise, a gel electrophoresis experiment revealed that NSGM **13** inhibited the formation of γ-γ polymers (~117 kD) at concentrations of 1000, 200, and 40 μM. In fact, it appears that inhibitor **13** dose-dependently inhibits the FXIIIa-mediated polymerization process, and therefore, the intensity (quantity) of α-, β-, γ-bands increased at least 2-fold as the concentration of inhibitor **13** increases from 40 μM to 1000 μM (see S1 Fig). Overall, inhibitor **13** was found to not only inhibit FXIIIa using dansylcadaverine and casein as substrates, but also it did inhibit the physiologic function of FXIIIa i.e. fibrin polymerization.

To confirm that this phenomenon was mainly because of action of inhibitor **13** on FXIIIa, the same experiment was repeated without adding either thrombin or FXIIIa. This resulted in formation of only about 24 ± 4% and 13.5 ± 1.5% turbidity, respectively, as compared to the control experiment. This implies that fibrin polymerization was inhibited due to the action of **13** on either thrombin or FXIIIa. We also evaluated the direct inhibition of human α-thrombin by **13** using the corresponding standard chromogenic substrate hydrolysis assay. NSGM **13** was found to not inhibit human α-thrombin at the highest concentration tested of 930 μM (Fig 3A), which implies at least 26-fold selectivity for FXIIIa. Thus, inhibitor **13** appears to inhibit the physiologic function of FXIIIa as measured by cross-linking of fibrin monomers. The *IC*_*50*_ of fibrin crosslinking inhibition was measured to be 76.3 μM with an efficacy value of ~100% under near physiologic conditions of pH 7.4 and 25°C (Fig 6B).

## Conclusions and Significance

For this study, we proposed that allosteric modulation of FXIIIa should be possible by targeting one or more of the anion-binding site(s) on the A subunit of FXIIIa. We found that heparin does bind to FXIIIa with a *K*_*D*_ value of 2.4 μM, yet it does not inhibit its function. On the contrary, NSGM **13** was found to moderately bind and inhibit human FXIIIa with a *K*_*D*_ of 25 μM and an *IC*_*50*_ of 36 μM. Allosteric inhibition of FXIIIa by **13** was suggested by Michaelis–Menten kinetics and this induced inhibition of FXIIIa–mediated fibrin polymerization with *IC*_*50*_ of 76 μM. Interestingly, similar molecule with the same flavonoid scaffold and number of sulfate groups i.e., inhibitor **14** only inhibited FXIIIa weakly with and an *IC*_*50*_ of 118 μM. Structurally, inhibitor **13** possesses two ethylene units bridging the three flavonoid moieties whereas inhibitor **14** possesses two propylene linkers. The increased flexibility of the latter inhibitor perhaps makes it less probable for the molecule to adopt an optimal three dimensional orientations of the sulfate and aromatic moieties which are better recognized by the anion-binding site(s) of human FXIIIa. This indicates that small structural differences have significant impact on the potency of flavonoid-based allosteric inhibitors of FXIIIa and suggests that further medicinal chemistry efforts should focus on optimizing the number and the position of sulfate groups to engineer higher potency in addition to higher selectivity.

This work shows for the first time that allosteric modulation of FXIIIa is achievable by exploiting the coupling between the heparin-binding site and enzyme’s active site. The interesting aspect of this modulation is that NSGMs are able to induce inhibition but not GAGs. In fact, it is this made even more interesting when one takes into account that heparin binding to FXIIIa appears to be a cooperative process, as evidenced by the sigmoidal binding profile (n = 1.9 ± 0.6; see Fig 3C). This implies that cooperativity does not automatically induce inhibition, i.e., not enough change in the structure of the active site. On the other hand, NSGM **13** interaction with FXIIIa appears to be a non-sigmoidal binding process, although inducing allosteric inhibition. The fundamental reason(s) for these differences remain to be understood.

The presence of aromatic/hydrophobic character in a FXIIIa ligand is likely to be critical. This aspect has also been observed earlier for inhibition of thrombin [37, 38, 48] and factor XIa [50]. As stated in an editorial some time ago [49], the combination of hydrophilic/negative charge and aromatic/hydrophobic character in NSGMs introduces significant functional differences from the molecules that attempt to mimic–GAGs. These differences appear to arise from the interaction of NSGMs with non-basic residues in addition to lysines and arginines, which help originate an alternate network of coupled residues resulting in an alternate allosteric conformational change. Recent results suggest that multiple such networks exist in thrombin [51, 52], a highly plastic coagulation factor. This work shows that FXIIIa, despite being a completely different type of enzyme from typical serine proteases of the coagulation cascade, exhibits similar allosterism.

In principle, allosteric inhibition of coagulation factors can induce homeostasis, a state wherein pro-coagulation and anti-coagulation propensities are perfectly balanced [53]. Such a state has not been possible to achieve with any of the orthosteric inhibitors, which is the primary reason for adverse consequences associated with all current drugs [5]. Achieving homeostasis is challenging and NSGM **13** certainly does not display this property, as evidenced by the nearly 100% inhibition of FXIIIa at saturation. However, the first group of NSGMs that point to the possibility of inducing homeostasis have been devised for thrombin [37, 38, 48], which suggest that it may be possible to design/discover such agents for FXIIIa.

Allosteric inhibitors offer several advantages over orthosteric inhibitors [24–27]. Allosteric inhibitors tend to be more specific in recognition and function as allosteric sites of enzymes belonging to the same superfamily tend to be less conserved as compared to their more similar active sites. In principle therefore, allosteric inhibitors afford a better opportunity to develop safer therapeutics. NSGM **13** does not exhibit this level of specificity, as evidenced by the similarity of its inhibition of transglutaminase. Yet, it is able to select FXIIIa over thrombin, factor Xa, and the cysteine protease papain. We predict that computational molecular modeling aided by co-crystal structures of the FXIIIa–**13** and transglutaminase–**13** complexes would help design advanced agents that more selectively target FXIIIa.

Despite the interesting mechanistic aspect of NSGM **13**, it is important to emphasize that this study has been conducted in an isolated/purified system to establish the promising phenomenon of allosteric modulation of FXIIIa’s enzymatic activity by targeting a putative anion-binding site. Effect of this molecule on the whole coagulation process as determined in human plasma or whole blood remains to be thoroughly investigated.

Overall, our work has presented initial evidence that an alternative approach to modulate FXIIIa through allosterism is clearly possible, very promising and likely to engineer molecules with reduced bleeding risks. Furthermore, the use of specific NSGMs to study mechanism of action may enhance our understanding of the role of FXIIIa in hemostasis. Finally, this work also suggests the possibility of NSGM-mediated allosteric modulation of other transglutaminases, which may have implications in other physiologic and/or pathologic processes.

## Materials and Methods

### Chemicals, Reagents, Analytical Chemistry, Enzymes, and Substrates

All anhydrous organic solvents were purchased from Sigma-Aldrich (St Louis, MO) or Fisher (Waltham, MA) and used as such. Other solvents used were of reagent gradient and used as purchased. Analytical TLC was performed using UNIPLATE^TM^ silica gel GHLF 250 um pre-coated plates (ANALTECH, Newark, DE). Silica gel (200–400 mesh, 60 Å), fibrinogen, dansylcadaverine, dithiothreitol (DTT), and *N*,*N*-dimethylcasein were from Sigma-Aldrich. Chemical reactions sensitive to air or moisture were carried out under nitrogen atmosphere in oven-dried glassware. Reagent solutions, unless otherwise noted, were handled under a nitrogen atmosphere using syringe techniques. Flash chromatography was performed using Teledyne ISCO (Lincoln, NE) Combiflash RF system and disposable normal silica cartridges of 30–50 μ particle size, 230–400 mesh size and 60 Å pore size. The flow rate of the mobile phase was in the range of 18 to 35 ml/min and mobile phase gradients of ethyl acetate/hexanes and CH_2_Cl_2_/CH_3_OH were used to elute compounds. Human FXIIIa, α-thrombin, and factor Xa were obtained from Haematologic Technologies (Essex Junction, VT). Stock solution of FXIIIa was prepared in 50 mM TrisHCl buffer, pH 8.0, containing 10 mM CaCl_2_ and 100 mM NaCl. Stock concentration of fibrinogen was prepared in 50 mM TrisHCl buffer, pH 7.4, containing 10 mM CaCl_2_. Stock solution of thrombin and factor Xa were prepared in 20 mM TrisHCl buffer, pH 7.4, containing 100 mM NaCl, 2.5 mM CaCl_2_, 0.1% PEG8000, and 0.02% Tween80. Chromogenic substrates of thrombin (Spectrozyme TH) and of factor Xa (Spectrozyme FXa) were obtained from Sekisui Diagnostics (Lexington, MA). Reagents for gel electrophoresis experiment were from ThermoFisher Scientific (Waltham, MA). Papain, its chromogenic substrate (*N*-α-benzoyl-*L*-arginyl-4-nitroanilide), and polybrene were all from Sigma-Aldrich. Papain was prepared in 50 mM TrisHCl buffer, pH 7.4, containing 100 mM NaCl, 100 mM DTT, 2.5 mM CaCl_2_, 0.1% PEG8000, and 0.02% Tween80.

### GAGs and NSGMs

All GAGs were obtained from commercial sources, while sulfated NSGMs were synthesized, as reported earlier [54, 55]. Of these, **13** was synthesized as depicted in S1 File. Briefly, the natural product quercetin **13a** was treated with 3–4 equivalents of MOM-Cl in the presence of strong base DIPEA at room temperature resulting in intermediates **13b** and **13c** with yields of 45% and 55%, respectively. The intermediate **13b** was regioselectively protected at positions-3, -7, and -4’. The presence of extra equivalent of MOM-Cl resulted in intermediate **13c**, which was protected at all phenolic groups except for position-5. Intermediate **13d** was then quantitatively prepared by base-mediated SN_2_ alkylation of **13c** using the same amount of dibromoethane. After that, 2.5 equivalents of K_2_CO_3_ was added to **13b** in DMF followed by the addition of 2 equivalents of intermediate **13d**, which led to the formation of per-protected trimeric flavone. The MOM groups were then deprotected by refluxing the per-protected intermediate in MeOH in the presence of catalytic amount of para-toluenesulfonic acid (*p*-TSA). Sulfation of the resulting phenolic precursor was achieved using microwave-assisted protocol in which a stirred solution of trimeric polyphenol in anhydrous CH_3_CN was treated with the base Et_3_N (10 equiv. per −OH group) and the sulfating agent SO_3_/Me_3_N complex (6 equiv. per −OH) at room temperature. The reaction mixture was microwaved for 8 h at 90°C resulting in the per-sulfated trimer **13**. The overall yield of the last three steps of coupling, deprotection, and sulfation was about 60% (see S1 File).

### Chemical Characterization of Molecules

^1^H and ^13^C NMR were recorded on Bruker-400 MHz spectrometer in either CD_3_OD, CDCl_3_, acetone-*d*_*6*_, D_2_O, or DMSO-*d*_*6*_. Signals, in part per million (ppm), are either relative to the internal standard or to the residual peak of the solvent. The NMR data are reported as chemical shift (ppm), multiplicity of signal (s = singlet, d = doublet, t = triplet, q = quartet, dd = doublet of doublet, m = multiplet), coupling constants (Hz), and integration. ESI-MS of compounds were recorded using Waters Acquity TQD MS spectrometer in positive or negative ion mode. Samples were dissolved in methanol and infused at a rate of 20 μL/min. For HRMS measurements, a Perkin Elmer AxION 2 TOF MS was used in negative ion mode. Ionization conditions on both instruments were optimized for each compound to maximize the ionization of the parent ion. Final sulfated products (**1**–**15**) were obtained in high overall yields (>50%), had >95% purity, and their NMR and MS data were found to be identical to values reported earlier in literature [54, 55]. See S1 File.

### Direct Inhibition of Human FXIIIa, Thrombin, Factor Xa, and Papain by Sulfated Molecules

Direct inhibition of human FXIIIa was measured spectrofluorometrically by a bisubstrate, fluorescence-based transglutamination assay at pH 8.0 and 37°C on a microplate reader (FlexStation III, Molecular Devices) (modified from earlier reports) [47, 56]. Briefly, to each well of a 96-well microplate containing 80 μL of 50 mM TrisHCl buffer, pH 8.0, containing 100 mM NaCl, 10 mM CaCl_2_, and 2 mg/mL dimethylcasein was added 5 μL potential NSGM-based inhibitor (0–20 mM aqueous solution) or vehicle, 5 μL DTT (20 mM), and 5 μL enzyme (0.12–1.2 μM) at 37°C. After 5 min incubation, 5 μL of dansylcadaverine (2.5 mM) was rapidly added and the residual enzyme activity was measured from the initial rate of increase in RFU (λ_EM_ = 550 nm, λ_EX_ = 360 nm). Relative residual enzyme activity (Y) as a function of the concentration of sulfated molecule was fitted using logistic Eq 1 to obtain the potency (*IC*_*50*_), efficacy (*ΔY*) and Hill slope (*HS*) of inhibition. In this equation, *Y*_*M*_ and *Y*_*o*_ are the maximal and minimal values of *Y*.

$$Y=Y_{0}+\frac{Y_{M}-Y_{0}}{1+10^{\left(log{\left[Inhibitor\right]}_{0}-log{IC}_{50})(HS\right)}}$$ (1)

Direct inhibition of thrombin, a GAG–binding protein, was measured using a chromogenic substrate hydrolysis assay on a microplate reader (FlexStation III, Molecular Devices), as reported earlier [37, 38]. Briefly, to each well of a 96-well microplate containing 185 μL of 20 mM TrisHCl buffer, pH 7.4, containing 100 mM NaCl, 2.5 mM CaCl_2_, 0.1% PEG8000, and 0.02% Tween80 was added 5 μL of inhibitor **13** (0–20 mM) or vehicle, and 5 μL of thrombin (240 nM) at 25°C. After 10 min incubation, 5 μL of Spectrozyme TH (2 mM) was rapidly added and the residual enzyme activity was measured from the initial rate of increase in A405. Relative residual enzyme activity as a function of the inhibitor concentration was measured and inhibition parameters were calculated using logistic Eq 1. Likewise, inhibition of factor Xa, another GAG–binding protein, by inhibitor **13** was performed in a similar fashion using the corresponding chromogenic substrate assay in pH 7.4 TrisHCl buffer at 37°C. The effective concentrations of factor Xa and its substrate (Spectrozyme FXa) were 43.5 nM and 5 mM, respectively.

Direct inhibition of papain, a cysteine protease, by inhibitor **13** was also evaluated using a chromogenic substrate assay on a microplate reader (FlexStation III, Molecular Devices) as reported earlier [15]. Briefly, to each well of a 96-well microplate containing 91 μL of 50 mM TrisHCl buffer, pH 7.4, containing 100 mM NaCl, 100 mM DTT, 2.5 mM CaCl_2_, 0.1% PEG8000, and 0.02% Tween80 was added 5 μL of inhibitor **13** (0–20 mM) or vehicle, and 3 μL of papain (130 U/mL) at 37°C. After 5 min incubation, 1 μL of *N-*α-benzoyl-*L*-arginine 4-nitroanilide hydrochloride, the chromogenic substrate (17.7 mM) was rapidly added and the residual enzyme activity was measured from the initial rate of increase in absorbance at 405 nm. Relative residual enzyme activity as a function of the inhibitor concentration was measured and inhibition parameters were calculated using logistic Eq 1.

### Fluorescence–Based Binding Affinity of Inhibitor 13 and UFH to Human FXIIIa

Fluorescence experiments were performed using a QM4 spectrofluorometer (Photon Technology International, Birmingham, NJ) in 50 mM TrisHCl buffer, pH 7.4, containing 100 mM NaCl and 10 mM CaCl_2_ at 37°C. The affinity of FXIIIa for either inhibitor **13** or UFH was measured using the change in the intrinsic tryptophan fluorescence (λ_EM_ = 348 nm, λ_EX_ = 280 nm) at varying concentrations of the ligand [L]. The titrations were performed by adding aliquots of 250 μM aqueous solution of inhibitor **13** or UFH to 200 μL solution of FXIIIa (127 nM) and monitoring the fluorescence intensity at the appropriate λ_EM_. The excitation and emission slits were set to 1.0 mm. The observed change in fluorescence (*ΔF*) relative to initial fluorescence (*F*_*o*_) was fitted using Eq 2 (inhibitor **13**) or Eq 3 (UFH) to obtain the dissociation constant (*K*_*D*_) and the maximal change in fluorescence (*ΔF*_*MAX*_) at saturation. In Eq 3, the Hill coefficient “n” is a measure of the cooperativity of binding. Each measurement was performed three times.

$$\frac{\Delta F}{F_{0}}=\frac{{\Delta F}_{MAX}}{F_{0}}\times \frac{({\left[FXIIIa\right]}_{0}+{\left[13\right]}_{0}+K_{D})-\sqrt{{\left({\left[FXIIIa\right]}_{0}+{\left[13\right]}_{0}+K_{D}\right)}^{2}-4{\left[FXIIIa\right]}_{0}{\left[13\right]}_{0}}}{2{\left[FXIIIa\right]}_{0}}$$ (2)

$$\frac{\Delta F}{F_{o}}=\Delta F_{MAX}\times \frac{{\left[UFH\right]}^{n}}{({K_{D})}^{n}+{\left[UFH\right]}^{n}}$$ (3)

### Michaelis–Menten Kinetics of Dansylcadaverine and *N*,*N*-Dimethylcasein Conjugation Rate by Human FXIIIa

The initial rate of dansylcadaverine and *N*,*N*-dimethylcasein conjugation by FXIIIa was obtained from the linear increase in fluorescence at λ_EM_ = 550 nm (λ_EX_ = 360 nm). The initial rate was measured as a function of various concentrations of 1) dansylcadaverine (0–15 mM; effective concentrations in the well were 0−750 μM) at fixed saturating concentration of dimethyl-casein (5 mg/mL) or 2) dimethylcasein (0–5 mg/mL) at fixed concentration of dansylcadaverine (5 mM; effective concentration was 250 μM) in the absence or presence of inhibitor **13** (0, 8, 40, or 120 μM) in 50 mM TrisHCl buffer, pH 8.0, containing 100 mM NaCl, 1 mM DTT, and 10 mM CaCl_2_ at 37°C. The data was fitted using the standard Michaelis−Menten Eq 4 to determine the *K*_*M*_ and *V*_*MAX*_.

$$V_{i}=\frac{V_{MAX}\times [S]}{K_{M}+[S]}$$ (4)

### Polybrene Reversibility of FXIIIa Inhibition by NSGM 13

To assess the *in vitro* reversibility of FXIIIa inhibition, the activity profiles were measured in the presence of increasing concentrations of polybrene and inhibitor **13** (100 μM) at pH 7.4 and 37°C. Generally, each well of the 96-well microplate contained 80 μL of 50 mM TrisHCl buffer of pH 8.0 containing 100 mM NaCl, 1 mM DTT, 10 mM CaCl_2_, and 2 mg/mL dimethylcasein to which 5 μL of inhibitor **13** (2 mM) or vehicle, 5 μL of FXIIIa (stock of 0.6 μM), and 5 μL of prolybrene (0–60 mg/mL; effective concentrations were 0–3 mg/mL) were sequentially added. After a 5 min incubation, 5 μL of dansylcadaverine (2.5 mM; effective concentration in the well was 125 μM) was rapidly added and the restored FXIIIa activity was measured from the initial rate of increase in fluorescence at λ_EM_ = 550 nm (λ_EX_ = 360 nm). Stock of polybrene was serially diluted to give 7 different aliquots in the wells. Relative restored FXIIIa activity at each concentration of polybrene was calculated from the ratio of FXIIIa activity in the presence and absence of the reversing agent. Eq 5 was used to fit the dose dependence of restored transglutaminase activity to obtain the effective concentration of reversing agent required to restore 50% of enzyme activity at specific inhibitor concentration (*EC*_*50*_) and the efficacy (*ΔY*) of reversing process.

$$Y=Y_{0}+\frac{Y_{M}-Y_{0}}{1+10^{\left(log{\left[polybrene\right]}_{0}-log{EC}_{50})(-HS\right)}}$$ (5)

### Effect of Inhibitor 13 on FXIIIa-Mediated Fibrin Polymerization

This effect was determined by measuring the absorbance of an aqueous mixture of fibrinogen, human FXIIIa, and α-thrombin at 25°C on a microplate reader (FlexStation III, Molecular Devices). This experiment is a modified platform of a standard turbidity test [15, 17]. In this test, a 170 μL of the above mixture was formed by mixing 1) a 60 μL solution of fibrinogen (10 mg/mL) and human FXIIIa (2 μg/mL) in 50 mM TrisHCl buffer, pH 7.4, containing 10 mM CaCl_2_, 2) a 30 μL solution of inhibitor **13** (5, 50, or 500 μM) or vehicle, and 3) an 80 μL solution of thrombin (2.5 μg/mL) in 50 mM TrisHCl buffer, pH 7.4, containing 10 mM CaCl_2_. After 15 min incubation, the absorbance was measured at 405 nm. Same exercise was repeated without adding human α-thrombin or FXIIIa. The experiment was also performed at 24 different concentrations of inhibitor **13** (0–1177 μM). At each concentration, the relative residual FXIIIa activity causing fibrin polymerization (as indicated by turbidity) was determined by measuring the well absorbance at 405 nm. Relative residual enzyme activity (Y) as a function of the concentration of inhibitor **13** was fitted using logistic Eq 1 to obtain the *IC*_*50*_, *ΔY*, and *HS* of inhibition.

The effect of inhibitor **13** on fibrin polymerization was further investigated by gel electrophoresis as reported earlier [15] (See S1 Fig). A solution containing 13 mg/ml fibrinogen and 2.0 μg/mL FXIIIa (in the aforementioned TrisHCl buffer of pH 7.4 containing 10 mM CaCl_2_) was clotted in the presence and absence of human α-thrombin (2.5 μg/mL). The resulting mixture was either incubated with inhibitor **13** (1000, 200, and 40 μM) or buffer. The clots were incubated for 24 hrs at room temperature before the addition of denaturing buffer of 25 mM NaH_2_PO_4_, 5.7 M urea, 1.9% (w/v) SDS and 1.9% (w/v) DTT and then incubated overnight at 25°C. Samples were boiled in a water bath for 10 min before centrifugation at 12 000 g at 20°C for 3 min. The supernatants were examined by SDS/PAGE on homogeneous 7.5% cross-linked gels and stained with Coomassie Brilliant Blue followed by silver stain.

## Supporting Information

S1 Fig Evaluation of FXIIIa-mediated fibrin polymerization by gel electrophoresis in the presence of inhibitor 13. The gel electrophoresis experiment shows a dose-dependent effect of inhibitor **13** (1000, 200, and 40 μM) on fibrin cross-linking.
(PDF)

S1 File Synthesis and characterization of NSGM 13. Synthetic scheme and synthetic protocols for inhibitor **13** and its precursors are provided. Characterization data (^1^H and ^13^C NMR, and MS/ESI) of the flavonoid trimer **13** and its precursors are also provided.
(PDF)

S1 Table Evaluation of enzyme concentration effect on NSGM 13 inhibition parameters. Inhibition parameters (*IC*_*50*_, *HS*, and *ΔY*) of inhibitor **13** toward human FXIIIa using different enzyme concentrations (0, 6, 18, and 30 nM).
(PDF)
