# Supplementary material for: Allosteric Inhibition of Factor XIIIa. Non-Saccharide Glycosaminoglycan Mimetics, but Not Glycosaminoglycans, Exhibit Promising Inhibition Profile
Source: PLoS One. 2016 Jul 28;11(7):e0160189. doi: 10.1371/journal.pone.0160189 (PMC4965010; doi:10.1371/journal.pone.0160189)
Supplement: S1 Fig — The gel electrophoresis experiment shows a dose-dependent effect of inhibitor 13 (1000, 200, and 40 μM) on fibrin cross-linking. (PDF) [file pone.0160189.s001.pdf]

Supplementary Information

Allosteric Inhibition of Factor XIIIa. Non-Saccharide Glycosaminoglycan Mimetics, but not Glycosaminoglycans, Exhibit Promising Inhibition Profile

Rami A. Al-Horani, Rajesh Karuturi, Michael Lee, Daniel K Afosah, and Umesh R. Desai*

Department of Medicinal Chemistry
&
Institute for Structural Biology, Drug Discovery and Development
Virginia Commonwealth University

S1 Figure. Evaluation of FXIIIa-mediated fibrin polymerization by gel electrophoresis in the presence of inhibitor 13. The gel electrophoresis experiment shows a dose-dependent effect of inhibitor **13** (1000, 200, and 40 µM) on fibrin cross-linking.

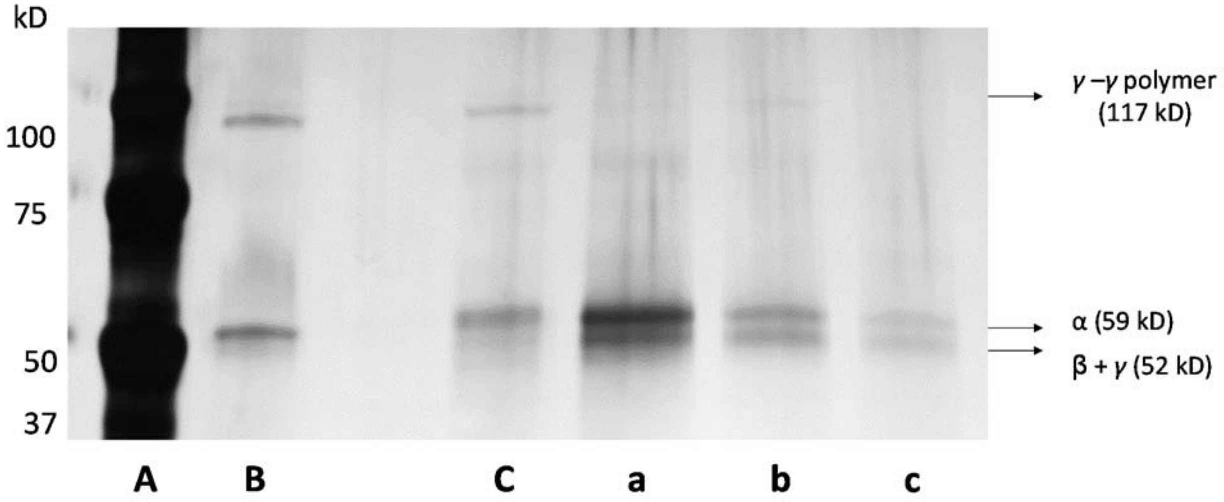

The effect of inhibitor **13** on fibrin polymerization was further investigated by gel electrophoresis as reported earlier in ref. 15 for tridegin. A solution containing 13 mg/ml fibrinogen and 2.0 µg/mL FXIIIa (in the aforementioned TrisHCl buffer of pH 7.4 containing 10 mM $CaCl_2$) was clotted in the presence and absence of human α-thrombin (2.5 µg/mL). The resulting mixture was either incubated with inhibitor **13** (1000, 200, or 40 µM) or buffer. The clots were incubated for 24 hrs at room temperature before the addition of denaturing buffer of 25 mM NaH_2PO_4, 5.7 M urea, 1.9% (w/v) SDS and 1.9% (w/v) DTT and then incubated overnight at 25 °C. Samples were boiled in a water bath for 10 min before centrifugation at 12 000 g at 20 °C for 3 min; the supernatants were examined by SDS/PAGE on homogeneous 7.5 % cross-linked gels and stained with Coomassie Brilliant Blue followed by silver stain.

Lane A, protein markers (Bio-Rad); Lane B, cross-linked fibrin formed in the presence of 45 µL (13 mg/mL fibrinogen + 2 µg/mL human FXIIIa) and 50 µL human α-thrombin in pH 7.4 TrisHCl buffer. The lane shows the γ- and β-bands (~52 kDa), which overlap, the α-band (~59 kDa) and the cross-linked proteins, γ-γ polymers (~117 kDa); Lane C, fibrinogen and FXIIIa incubated without thrombin showing the positions of the γ- (~52 kDa), Bβ- (~58 kDa) and Aα- (~65 kDa) bands as well as γ-γ polymers (~117 kDa); Lane a, the mixture of fibrinogen (13 mg/mL), FXIIIa (2.0 µg/mL), and α-thrombin (2.5 µg/mL) incubated with 1000 µM of inhibitor **13** showing a highly dense bands of α-, β-, and γ-bands with no band corresponds to γ-γ-polymers; Lane b, the mixture of fibrinogen (13 mg/mL), FXIIIa (2.0 µg/mL), and α-thrombin (2.5 µg/mL) incubated with 200 µM of inhibitor **13** showing a lesser dense bands of α-, β-, and γ-bands with no significant band corresponding to γ-γ-polymers; Lane c, the mixture of fibrinogen (13 mg/mL), FXIIIa (2.0 µg/mL), and α-thrombin (2.5 µg/mL) incubated with 40 µM of inhibitor **13** showing a lesser dense bands of α-, β-, and γ-bands with no significant band corresponding to γ-γ-polymers. The gel does not show the α-polymers (>~209 kDa) which typically form slowly and the majority of which are too large to enter the gel.
