# Supplementary material for: Allosteric Inhibition of Factor XIIIa. Non-Saccharide Glycosaminoglycan Mimetics, but Not Glycosaminoglycans, Exhibit Promising Inhibition Profile
Source: PLoS One. 2016 Jul 28;11(7):e0160189. doi: 10.1371/journal.pone.0160189 (PMC4965010; doi:10.1371/journal.pone.0160189)
Supplement: S1 File — Synthetic scheme and synthetic protocols for inhibitor 13 and its precursors are provided. Characterization data (1H and 13C NMR, and MS/ESI) of the flavonoid trimer 13 and its precursors are also provided. (PDF) [file pone.0160189.s002.pdf]

Supplementary Information

Allosteric Inhibition of Factor XIIIa.
Non-Saccharide Glycosaminoglycan Mimetics, but not Glycosaminoglycans, Exhibit Promising Inhibition Profile

Rami A. Al-Horani, Rajesh Karuturi, Michael Lee, Daniel K Afosah, and Umesh R. Desai*

Department of Medicinal Chemistry
&
Institute for Structural Biology, Drug Discovery and Development
Virginia Commonwealth University

S1 File. Synthesis and characterization of NSGM 13. Synthetic scheme and synthetic protocols for inhibitor **13** and its precursors are provided. Characterization data (^{1}H and ^{13}C NMR, and MS/ESI) of the flavonoid trimer **13** and its precursors are also provided.

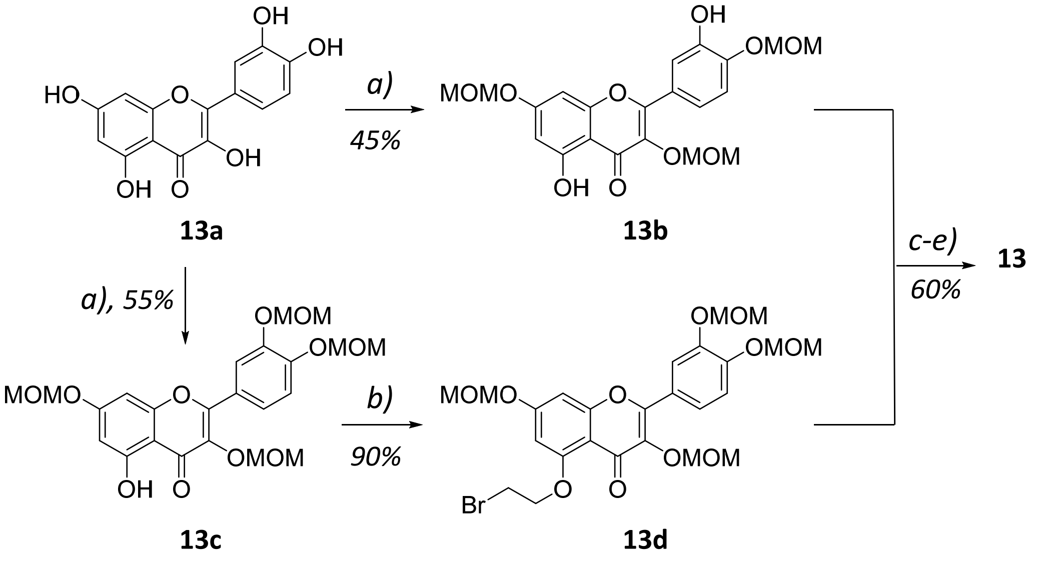

Scheme 1. Chemical synthesis of NSGM **13**. *a)* MOM-Cl (3–4 eq.), DIPEA, DCM, rt, 12 h, 45-55%; *b)* K_2CO_3 (1.1 eq), dibromoethane (1.1 eq), DMF, rt, 4 h, 90%; *c)* K_2CO_3 (2.2 eq), DMF, 60 °C, 4 h; *d) p*-TSA, MeOH, reflux, 48 h; *e)* SO_3/Me_3N (6 eq/-OH), Et_3N (10 eq/-OH), CH_3CN, microwave, 90 °C, 8 h, 60% (last three steps).

2-(3,4-bis(methoxymethoxy)phenyl)-5-(2-bromoethoxy)-3,7-bis(methoxymethoxy)-4Hchromen-4-one (13d). ^{1}H NMR (400 MHz, $CDCl_3$): 7.69 (d, 1 H, *J* = 2.0 Hz), 7.55-7.52 (m, 1 H), 7.09 (d, 1 H, *J* = 8.6 Hz), 6.54 (d, 1 H, *J* = 2.1 Hz), 6.27 (d, 1 H, d, *J* = 2.1 Hz), 5.13 (s, 2 H), 5.09 (s, 2 H), 5.05 (s, 2 H), 5.04 (s, 2 H), 4.37 (t, 2 H, *J* = 2.8 Hz), 3.51 (t, 2 H, *J* = 2.8 Hz),

3.39 (s, 3 H), 3.37 (s, 3 H), 3.34 (s, 3 H), 3.30 (s, 3 H). ^{13}C NMR (100 MHz, $CDCl_3$): 173.80, 161.47, 161.08, 158.57, 153.19, 149.03, 147.02, 137.75, 124.73, 123.28, 115.82, 115.48, 109.93, 97.58, 96.59, 95.33, 95.25, 94.36, 78.30, 76.04, 57.67, 57.10, 56.44, 56.41, 55.93. ESI-MS calculated for $C_{25}H_{29}BrO_{11}$ $[(M+H)]^+$, m/z, 585.07 found m/z 585.026.

Per-MOM protected flavonoid trimer. ^{1}H NMR ($CDCl_3$, 400 MHz): 7.83-7.82 (m, 2 H), 7.71 (d, *J* = 2.0 Hz, 1 H), 7.64-7.59 (m, 3 H), 7.19-7.14 (m, 3 H), 6.68-6.55 (m, 6 H), 5.22-5.20 (m, 10 H), 5.19 (s, 4 H), 5.13 (s, 4 H), 5.09 (s, 4 H), 4.54-4.46 (m, 8 H), 3.47-3.46 (m, 18 H), 3.43 (s, 6 H), 3.16 (s, 3 H), 3.15 (s, 3 H), 3.14 (s, 3 H). ^{13}C NMR (DMSO-d_6, 100 MHz): 173.57, 173.39, 161.45, 161.25, 160.10, 159.96, 158.46, 153.40, 153.30, 149.26, 149.0, 148.62, 146.65, 137.93, 125.20, 125.04, 124.99, 123.7, 123.09, 117.91, 116.69, 115.28, 110.72, 110.54, 99.59, 97.90, 97.82, 97.72, 96.47, 95.77, 95.63, 95.24, 94.37, 68.29, 67.89, 57.61, 57.54, 56.40, 56.30. ESI-MS calculated for $C_{71}H_{78}O_{32}$ $[(M+H)]^+$, m/z 1443.36, found $[(M+H)]^+$, m/z 1443.187.

Polyphenolic precursor of 13. ^{1}H NMR (DMSO-d_6, 400 MHz): 10.69 (bs, 3 H), 9.53-8.7 (bs, 6 H), 7.83 (d, *J* = 0.1 Hz, 1 H), 7.70-7.64 (m, 3 H), 7.49 (d, *J* = 9.0 Hz, 2 H), 6.98 (d, *J* = 8.6 Hz, 1 H), 6.87 (d, *J* = 8.5 Hz, 2 H), 6.59 (d, J = 1.9 Hz, 1 H), 6.51-6.44 (m, 5H), 4.45-4.40 (m, 8 H). ^{13}C NMR (DMSO-d_6, 100 MHz): 170.92, 170.81, 162.38, 162.34, 159.47, 157.94, 157.89, 148.61, 146.95, 146.26, 145.04, 141.98, 141.92, 141.63, 137.24, 137.24, 137.12, 137.09, 127.99, 125.47, 122.42, 121.49, 119.11, 115.58, 114.51, 113.50, 105.52, 105.44, 97.71, 95.20, 95.10, 67.84, 67.72, 59.69. ESI-MS calculated for C49H34O21 $[(M+H)]^+$, m/z 958.16, found $[(M+H)]^+$, m/z 958.187.

(13). ^{1}H NMR (DMSO-d_6, 400 MHz): 8.18-8.06 (m, 5 H), 7.79-7.63 (m, 4 H), 7.31-7.14 (m, 3 H), 6.92-6.74 (m, 3 H), 4.52 (s, 6 H), 4.42 (s, 2 H). ^{13}C NMR (DMSO-d_6, 100 MHz): 170.92, 170.81, 162.38, 162.34, 159.47, 157.94, 157.89, 148.61, 146.95, 146.26, 145.04, 141.98, 141.92, 141.63, 137.24, 137.12, 137.09, 127.99, 125.47, 122.42, 122.26, 121.49, 119.11, 115.84, 115.58, 114.51, 113.50, 105.52, 105.44, 97.71, 95.20, 95.10, 67.84, 67.72, 59.69. ESI-MS calculated for $C_{49}H_{23}Na_{11}O_{54}$S11 $[(M+Na)]^+$, m/z 2104.28, found $[(M\text{-}11Na+11\ HxA)+2HxA]^{2+}$, m/z 1578.349.
