# Supplementary material for: Allosteric Inhibition of Factor XIIIa. Non-Saccharide Glycosaminoglycan Mimetics, but Not Glycosaminoglycans, Exhibit Promising Inhibition Profile
Source: PLoS One. 2016 Jul 28;11(7):e0160189. doi: 10.1371/journal.pone.0160189 (PMC4965010; doi:10.1371/journal.pone.0160189)
Supplement: S1 Table — Inhibition parameters (IC50, HS, and ΔY) of inhibitor 13 toward human FXIIIa using different enzyme concentrations (0, 6, 18, and 30 nM). (PDF) [file pone.0160189.s003.pdf]

Supplementary Information

Allosteric Inhibition of Factor XIIIa.
Non-Saccharide Glycosaminoglycan Mimetics, but not Glycosaminoglycans, Exhibit Promising Inhibition Profile

Rami A. Al-Horani, Rajesh Karuturi, Michael Lee, Daniel K Afosah, and Umesh R. Desai*

Department of Medicinal Chemistry
&
Institute for Structural Biology, Drug Discovery and Development
Virginia Commonwealth University

S1 Table. Evaluation of enzyme concentration effect on NSGM 13 inhibition parameters. Inhibition parameters (IC_{50}, HS, and ΔY) of inhibitor **13** toward human FXIIIa using different enzyme concentrations. The incorporation of dansylcadverine (2.5 mM) onto dimethylcasein was measured at 37 °C in 50 mM TrisHCl, pH 8.0, buffer containing human FXIIIa [0; 6 nM; 18 nM; and 30 nM], 100 mM NaCl, 1 mM DTT, and 10 mM $CaCl_2$ at various concentrations of inhibitor **13**. No change in IC_{50}, *HS*, or *ΔY%* was observed at different enzyme concentrations as follows:

[FXIIIa] (nM)	IC_{50} (μM)	HS	ΔY (%)	Data point
**0**	*NA*[a]	*NA*	*NA*	6
**6**	45.5 ± 7.3	1.1 ± 0.4	103 ± 14	6
**18**	37.2 ± 2.0	1.3 ± 0.2	105 ± 5	6
**30**	36.2 ± 4.5	1.0 ± 0.3	107 ± 11	12

[a] Not available.
